# Impact of Leisure-Time Physical Activity on Glycemic Control and Cardiovascular Risk Factors in Japanese Patients with Type 2 Diabetes Mellitus: The Fukuoka Diabetes Registry

**DOI:** 10.1371/journal.pone.0098768

**Published:** 2014-06-04

**Authors:** Shinako Kaizu, Hiro Kishimoto, Masanori Iwase, Hiroki Fujii, Toshiaki Ohkuma, Hitoshi Ide, Tamaki Jodai, Yohei Kikuchi, Yasuhiro Idewaki, Yoichiro Hirakawa, Udai Nakamura, Takanari Kitazono

**Affiliations:** 1 Department of Medicine and Clinical Science, Graduate School of Medical Sciences, Kyushu University, Fukuoka, Japan; 2 Department of Environmental Medicine, Graduate School of Medical Sciences, Kyushu University, Fukuoka, Japan; 3 Diabetes Center, Hakujyuji Hospital, Fukuoka, Japan; 4 Division of General Internal Medicine, School of Oral Health Science, Kyushu Dental University, Kitakyushu, Japan; INSERM/UMR 1048, France

## Abstract

**Aims/hypothesis:**

The effects of leisure-time physical activity (LTPA) on glycemia and cardiovascular risk factors are not fully understood in Asian type 2 diabetic patients, who are typically non-obese. We studied associations between LTPA and glycemia and cardiovascular risk factors in Japanese type 2 diabetic patients.

**Methods:**

A total of 4,870 Japanese type 2 diabetic patients aged ≥20 years were divided into eight groups according to their LTPA. We investigated associations between the amount and intensity levels of physical activity (PA) and glycemic control, insulin sensitivity, cardiovascular risk factors, and low-grade systemic inflammation in a cross-sectional study.

**Results:**

LTPA was dose-dependently associated with body mass index (BMI), waist circumference, hemoglobin A_1c_ (HbA_1c_), fasting plasma glucose, homeostasis model assessment of insulin resistance, triglyceride, high density lipoprotein cholesterol, high sensitivity C-reactive protein, and prevalence of metabolic syndrome, but not with blood pressure, low density lipoprotein cholesterol or adiponectin. The amount of PA required to lower HbA_1c_ was greater than that required to improve cardiovascular risk factors. LTPA was inversely associated with HbA_1c_ in non-obese participants but not in obese participants after multivariate adjustments for age, sex, duration of diabetes, current smoking, current drinking, energy intake, cardiovascular diseases, depressive symptoms, and treatment of diabetes. Higher-intensity LTPA, not lower-intensity LTPA was associated with HbA_1c_ after multivariate adjustments with further adjustment including BMI.

**Conclusions/interpretation:**

LTPA was dose-dependently associated with better glycemic control and amelioration of some cardiovascular risk factors in Japanese type 2 diabetic patients. In addition, increased higher-intensity LTPA may be appropriate for glycemic control.

## Introduction

Beginning with Dr. Elliott P. Joslin in the 1920s, exercise therapy has been one of a myriad of treatments for diabetes mellitus. Until recently, however, the physiology of exercise and its clinical implications have not been well understood. The acute and chronic effects of physical activity (PA) on glycemia, insulin action, and hemodynamics have been extensively studied [1]. In 2010, the American Diabetes Association (ADA) and American College of Sports Medicine (ACSM) [1] published a joint position statement titled “Exercise and type 2 diabetes” containing 295 references, of which only a few were studies from Asia, where type 2 diabetes is rapidly becoming a serious medical issue [2]. Because large clinical trials examining exercise therapy are usually difficult to maintain because of poor adherence, a number of smaller studies have been performed and preferentially used for recent meta-analyses [3]–[8]. The joint position statement of the ADA and ACSM recommended 150 min per week of moderate-to-vigorous PA for type 2 diabetic patients, primarily based on prevention studies in obese patients conducted in Western countries [1], [9]–[11]. The beneficial effects of PA on glycemic control, cardiovascular risk factors, body composition, and mental health have been reported [6]–[8], [12]. However, these effects have not been confirmed in Asian diabetic patients who are typically non-obese.

Although the amount of PA is critical for determining its effectiveness on glycemic control, adherence to regular exercise is low in clinical practice. The optimal PA intensity level for the treatment of type 2 diabetes is controversial. Although high-intensity PA may lead to better glycemic control than low-intensity PA in intervention studies [13], type 2 diabetic patients are often unable to practice high-intensity PA because of comorbidity and aging. Therefore, observational studies in real-world settings are required to investigate the clinical usefulness of PA. In this context, we used a multicenter hospital-based database of Japanese type 2 diabetic patients in the present study. We investigated associations between the amount and intensity levels of PA and glycemic control, insulin sensitivity, cardiovascular risk factors, and low-grade systemic inflammation. As corresponding markers, we measured hemoglobin A_1c_ (HbA_1c_), fasting plasma glucose, homeostasis model assessment 2 of insulin resistance (HOMA2-IR), low density lipoprotein (LDL) cholesterol, metabolic syndrome and its components, adiponectin and high sensitivity C-reactive protein (HS-CRP), which have been widely used in epidemiological studies [14]–[16]. We found dose-effect relationships between the amount of PA and glycemic control and cardiovascular risk factors, and that higher-intensity PA, not lower-intensity PA, was associated with glycemic control.

## Methods

### Study subjects

The Fukuoka Diabetes Registry is a multicenter prospective study designed to investigate the influence of modern treatments on the prognosis of diabetic patients regularly attending teaching hospitals certified by the Japan Diabetes Society or certified diabetologists' clinics in Fukuoka Prefecture, Japan (UMIN Clinical Trial Registry 000002627) [17]. A total of 5,131 diabetic patients aged 20 years or older were registered between April 2008 and October 2010. Exclusion criteria were: (1) patients with drug-induced diabetes or receiving corticosteroid treatment; (2) patients undergoing renal replacement therapy; (3) patients with serious diseases other than diabetes, such as advanced malignancy or decompensated liver cirrhosis; and (4) patients unable to visit a diabetologist regularly. After excluding 261 people with type 1 diabetes, the remaining 4,870 participants were enrolled in this cross-sectional study.

This study was conducted with the approval of the Kyushu University Institutional Review Board, and written informed consent was obtained from all participants.

### Assessment of PA

Using a self-administered questionnaire [18], the type, frequency (times per week or month), and duration (min) of leisure-time physical activity (LTPA), and the frequency (times per week) and duration (min) of walking or cycling for commuting PA were assessed. The compendium of PA was used to estimate the intensity of PA in terms of metabolic equivalents (METs) [19]. PA was quantified as the sum of activity time (converted to hours per week) multiplied by the METs for each activity (METs·h/w), and total leisure-time physical activity (T-LTPA) was expressed as the sum of commuting PA and LTPA. Participants were divided into eight groups according to their octile of T-LTPA: −1.4 (O1), 1.5–5.6 (O2), 5.7–9.8 (O3), 9.9–13.2 (O4), 13.3–18.8 (O5), 18.9–26.5 (O6), 26.6–37.8 (O7), and 37.9–(O8) METs·h/w. Because the proportion of participants performing low-intensity PA (<3.0 METs) and high-intensity PA (≥6.0 METs) were only 6% (n = 291) and 10% (n = 457), respectively, we used 3.6 METs, the mean METs of participants, as a cut-off value for categorizing PA into lower- and higher-intensity PA.

### Clinical evaluation and laboratory measurements

At baseline examinations, participants completed a self-administered questionnaire covering duration of diabetes, alcohol intake and smoking habits, energy intake, history of cardiovascular diseases, and treatment of diabetes. Alcohol intake and smoking habits were classified as either current use or not. The participants were categorized as taking oral hypoglycemic agents, insulin therapy or not. The dietary survey was conducted using a brief-type self-administered diet history questionnaire regarding food frequency of 58 items (BDHQ; Gender Medical Research Inc., Tokyo, Japan) [20]. The presence of depressive symptoms was assessed using the Center for Epidemiologic Studies Depression Scale [21], and participants who scored 16 or more out of 60 points were defined as having depressive symptoms. Body weight and height were measured, body mass index (BMI) was calculated as weight (kilograms) divided by height squared (meters squared), and obesity was defined as BMI≥25 kg/m^2^ for Japanese people [22]. Waist circumference at the umbilical level was measured by a trained staff member with the participant in the standing position. Blood was collected by venipuncture after an overnight fast (4,402 participants) or after breakfast (468 participants). The fed participants were excluded in statistical analyses for fasting plasma glucose, serum C peptide, HOMA2-IR, triglyceride, and metabolic syndrome. Assessments were performed at one central laboratory. HbA_1c_ was determined using high-performance liquid chromatography (Tosoh Corp., Tokyo, Japan), plasma glucose by the glucose oxidase method, serum C-peptide by chemiluminescent immunoassay (Kyowa Medex, Tokyo, Japan), serum adiponectin and HS-CRP by latex immunonephelometry (Mitsubishi Chemical Medience, Tokyo, Japan; Siemens Healthcare Diagnostics, Tokyo, Japan), serum high density lipoprotein (HDL) cholesterol and serum LDL cholesterol by enzymatic methods (Sekisui Medical, Tokyo, Japan), and triglyceride by enzymatic methods (Shino-Test Corp., Tokyo, Japan). Insulin resistance was estimated based on fasting glucose and C-peptide concentrations using the updated homeostasis model assessment calculator provided by the Oxford Center for Diabetes Endocrinology and Metabolism [23] and expressed as HOMA2-IR after excluding 587 participants with unacceptable levels of fasting plasma glucose (<3.0 mmol/L or >25 mmol/L) or C-peptide (<0.2 nmol/L or >3.5 nmol/L) [24]. Metabolic syndrome was diagnosed on the basis of the harmonized definition 2009 [25], i.e., the presence of at least two of the following four components: elevated waist circumference for Asians (waist circumference ≥90 cm in men and ≥80 cm in women), elevated triglyceride (≥1.68 mmol/L and/or current use of drugs for elevated triglyceride), reduced HDL cholesterol (<1.03 mmol/L for men and <1.29 mmol/L for women and/or current use of drugs for reduced HDL cholesterol), and elevated blood pressure (blood pressure ≥130/85 mmHg and/or current use of anti-hypertensive drugs).

### Statistical analysis

Linear trends for age, sex, BMI, waist circumference, duration of diabetes, lifestyle and dietary factors, proportion of participants with metabolic syndrome or depressive symptoms, and treatment of diabetes across the categories of T-LTPA were tested using the Jonckheere–Terpstra test and the Cochran–Armitage test, as appropriate. Log transformations for HbA_1c_, triglyceride, HS-CRP and adiponectin were used to normalize skewed data, and the results were expressed as geometric means and their standard error. Age-, sex-, or multivariate-adjusted regression analyses were performed to estimate the linear association of T-LTPA, higher-intensity LTPA or lower-intensity LTPA with BMI, waist circumference, HbA_1c_, HOMA2-IR, triglyceride, HDL cholesterol, and HS-CRP. In the multivariate-adjusted analysis, we included age, sex, duration of diabetes, current smoking habits, current drinking habits, total energy intake, cardiovascular diseases, depressive symptoms, and treatment of diabetes (model 1). BMI was added for further adjustment (model 2). Higher-intensity LTPA was included in the multivariate-adjusted regression analysis of lower-intensity LTPA and vice versa. The correlation coefficient between higher and lower LTPA was −0.054 by Spearman's correlation analysis. For estimating the effects of the intensity of LTPA, interactions between T-LTPA and the percentage of higher-intensity LTPA per T-LTPA on BMI, waist circumstance, HbA_1c_, HOMA2-IR, triglyceride, HDL cholesterol, and HS-CRP were examined by adding an interaction term to the statistical model. All analyses were performed using the SAS software package version 9.3 (SAS Institute Inc., Cary, NC, USA). A P value of <0.05 was considered statistically significant in all analyses.

## Results

Table 1 shows clinical characteristics of the studied participants according to the octile of T-LTPA. As T-LTPA increased, the percentage of higher-intensity LTPA (≥3.6 METs) per T-LTPA increased. The participants were older and less obese with an increase in T-LTPA. The proportions of male participants and duration of diabetes, current drinkers and dietary energy intake increased, whereas BMI, waist circumstance, the proportions of current smokers, and participants with cardiovascular disease or depressive symptoms decreased. Although the proportion of participants treated with oral hypoglycemic agents did not differ among groups, the proportion of those using insulin reduced as T-LTPA increased.

**Table 1:** Clinical characteristics of the studied participants according to the octile of T-LTPA.

	All	Octile of T-LTPA								P value^a^
		O1	O2	O3	O4	O5	O6	O7	O8	
n	4,870	609	603	623	606	608	670	544	607	
Range (METs h/w)	-	0–1.4	1.5–5.6	5.7–9.8	9.9–13.2	13.3–18.8	18.9–26.5	26.6–37.8	37.9–	
T-LTPA (METs h/w)	18.9±19.0	0.1±0.4	3.5±1.2	7.7±1.3	11.9±1.1	15.9±1.6	23.1±2.5	31.6±3.1	58.1±20.3	<0.001
Higher-intensity LTPA per T-LTPA (%)	50	6	36	48	59	55	61	68	66	<0.001
Age (years)	66±10	64±11	65±11	65±11	65±10	66±10	66±10	67±8	67±9	<0.001
Male (%)	57	57	48	49	57	54	60	63	69	<0.001
BMI (kg/m^2^)	23.8±3.8	24.8±4.3	24.6±4.0	24.2±3.9	23.8±4.2	23.4±3.6	23.2±3.4	23.1±3.3	23.2±3.3	<0.001
Obesity (%)	31	43	41	36	30	26	25	22	24	<0.001
Waist circumference (cm)	86±10	89±11	88±10	87±10	86±10	85±10	84±9	84±9	84±9	<0.001
Duration of diabetes (years)	16±11	15±11	16±10	16±10	16±10	16±11	16±11	17±10	17±11	0.003
Current smoker (%)	19	24	21	18	20	18	16	17	15	0.001
Current drinker (%)	39	36	35	37	41	39	41	43	42	0.044
Dietary energy intake (×10^3^, kJ/day)	7.1±2.1	6.9±2.2	6.8±2.1	6.9±2.0	6.7±2.0	7.1±2.0	7.2±2.1	7.1±2.0	7.5±2.2	<0.001
Cardiovascular diseases (%)	24	27	29	25	22	22	26	21	22	0.013
Depressive symptoms (%)	9	13	10	11	8	9	7	5	6	<0.001
Oral hypoglycemic agents (%)	55	53	55	52	59	56	55	55	52	ns
Insulin (%)	28	32	31	31	24	28	26	27	26	0.011

Values are expressed as mean ± SD or percentages.

Abbreviations: T-LTPA, total leisure-time physical activity; LTPA, leisure-time physical activity; METs, metabolic equivalents; BMI, body mass index.

a Continuous variables were tested by the Jonckheere-Terpstra test and categorical variables were tested by the Chochran-Armitage test.

Higher-intensity LTPA was defined as ≥3.6 METs. Obesity was defined as BMI≥25 kg/m^2^ for Japanese [22].

**Figure 1:**
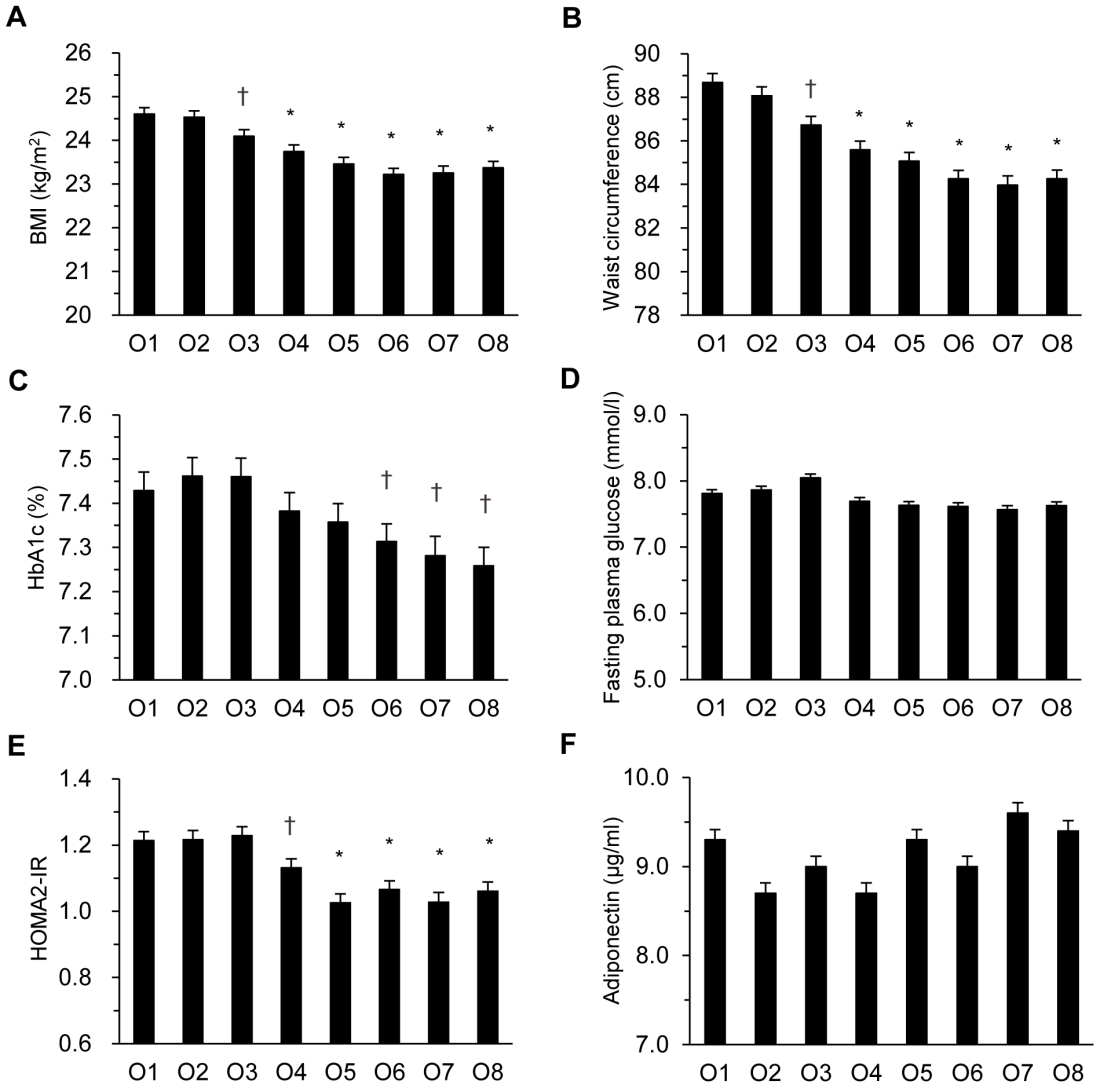
Changes in cardiovascular risk factors according to the octile of total leisure-time physical activity in Japanese type 2 diabetes patients. Age- and sex-adjusted body mass index (BMI) (A), waist circumference (B), hemoglobin A_1c_ (HbA_1c_) (C), fasting plasma glucose (D), homeostasis model assessment 2 of insulin resistance (HOMA2-IR) (E), and adiponectin (F) in the octile of T-LTPA in 4,870 Japanese type 2 diabetic patients. +p<0.05, *p<0.01 *vs.* the lowest O1.

**Figure 2:**
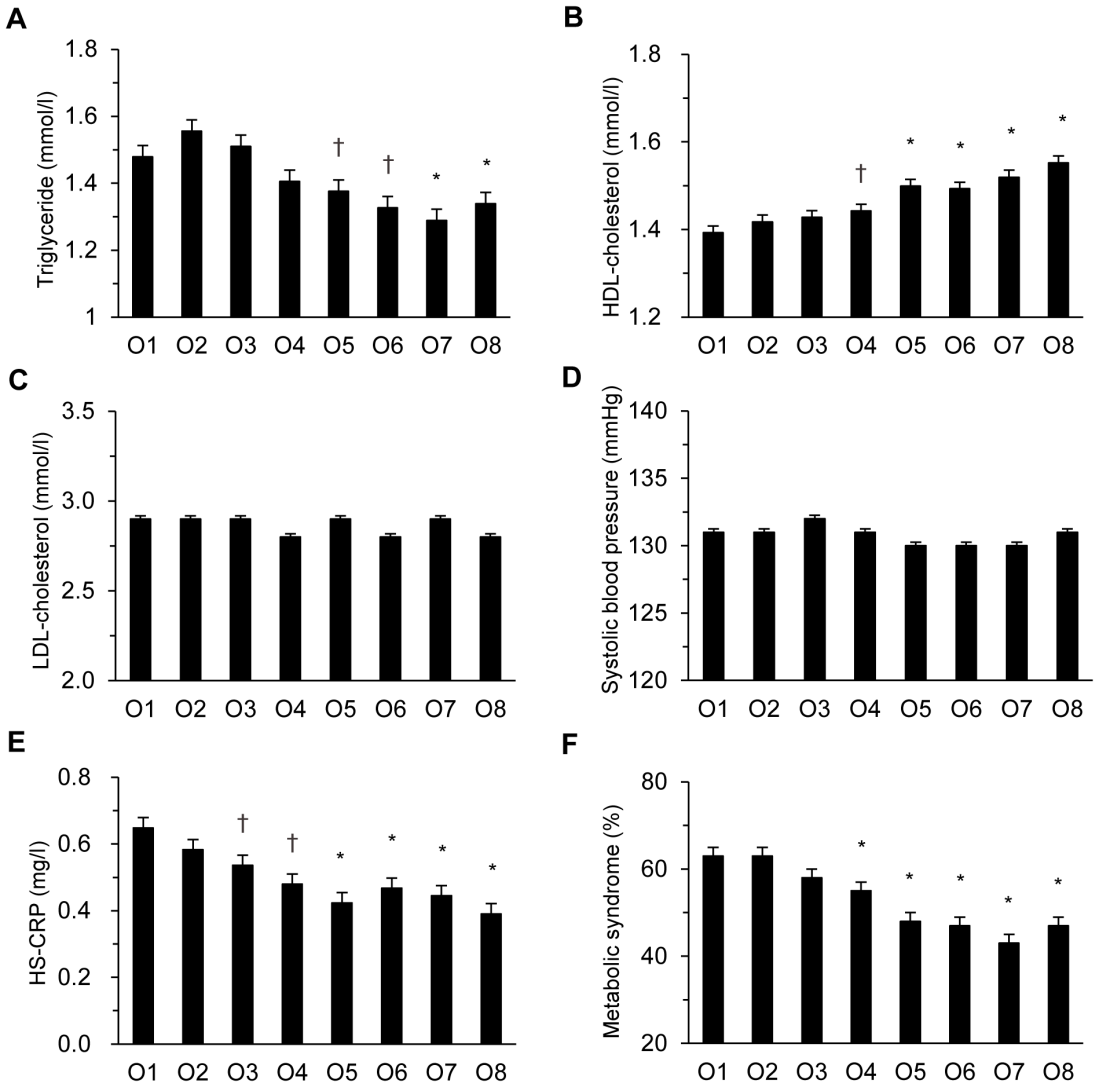
Changes in cardiovascular risk factors according to the octile of total leisure-time physical activity in Japanese type 2 diabetes patients. Age- and sex-adjusted triglyceride (A), high density lipoprotein (HDL) cholesterol (B), low density lipoprotein (LDL) cholesterol (C), systolic blood pressure (D), high sensitivity C-reactive protein (HS-CRP) (E), and prevalence of metabolic syndrome (F) in the octile of T-LTPA in 4,870 Japanese type 2 diabetic patients. +p<0.05, *p<0.01 *vs.* the lowest O1.

As shown in Figures 1 and 2, age- and sex-adjusted BMI, waist circumference, HbA_1c_, fasting plasma glucose, HOMA2-IR, triglyceride, HS-CRP and prevalence of metabolic syndrome decreased dose-dependently as T-LTPA increased (p for trend all <0.001 for BMI, waist circumference, HbA1c, fasting plasma glucose, HOMA2-IR, triglyceride, HS-CRP, and prevalence of metabolic syndrome). Age- and sex-adjusted HDL cholesterol increased dose-dependently with T-LTPA (p for trend <0.001). Compared with the lowest category O1, the difference became statistically significant at O3 for BMI, waist circumference, and HS-CRP, at O4 for HOMA2-IR, HDL cholesterol, and prevalence of metabolic syndrome, at O5 for triglyceride, and at O6 for HbA_1c_. Systolic blood pressure, LDL cholesterol, and serum adiponectin did not show a linear trend across octiles of T-LTPA.

Table 2 shows the results of multiple regression analysis of T-LTPA with metabolic and inflammatory variables in non-obese and obese participants. Waist circumference, HOMA2-IR, triglyceride, HDL cholesterol, and HS-CRP significantly correlated with T-LTPA in both non-obese and obese participants after multivariate adjustments for age, sex, duration of diabetes, current smoking, current drinking, energy intake, cardiovascular diseases, depressive symptoms, and treatment of diabetes. Conversely, T-LTPA significantly correlated with HbA_1c_ in non-obese participants but not in obese participants.

**Table 2:** Multiple regression analysis of T-LTPA with metabolic and inflammatory variables in non-obese and obese participants.

		Nonobese participants (n = 3,366)		Obese participants (n = 1,504)	
		Regression coefficient	p value	Regression coefficient	p value
		(per 1 MET h/w increase)		(per 1 MET h/w increase)	
Waist circumference (cm)	Model 1	−0.0313	<0.001	−0.0435	0.001
	Model 2	−0.0281	<0.001	−0.0391	0.003
HbA1c (%) (mmol/mol)	Model 1	−0.0003 (−0.0033)	0.002	−0.0004 (−0.0044)	0.073
	Model 2	−0.0002 (−0.0022)	0.027	−0.0002 (−0.0022)	ns
HOMA2-IR	Model 1	−0.0016	0.002	−0.0038	0.002
	Model 2	−0.0014	0.006	−0.0035	0.003
Triglyceride (mmol/l)	Model 1	−0.0023	<0.001	−0.0022	0.003
	Model 2	−0.0026	<0.001	−0.0018	0.013
HDL-cholesterol (mmol/l)	Model 1	0.0022	<0.001	0.0020	<0.001
	Model 2	0.0020	<0.001	0.0019	<0.001
HS-CRP (mg/l)	Model 1	−0.0044	<0.001	−0.0056	0.003
	Model 2	−0.0038	0.002	−0.0054	0.005

Abbreviations: T-LTPA, total leisure-time physical activity; METs, metabolic equivalents; HbA_1c_, hemoglobin A_1c_; HOMA2-IR, homeostasis model assessment 2 of insulin resistance; HDL, high density lipoprotein; HS-CRP, high sensitivity C-reactive protein; BMI, body mass index.

Obesity was defined as BMI≥25 kg/m^2^ for Japanese [22].

Model 1, adjusted for age and sex; Model 2, adjusted for age, sex, duration of diabetes, current smoking, current drinking, energy intake, cardiovascular diseases, depressive symptoms, and treatment of diabetes.

Table 3 shows the results of multiple regression analysis of lower- or higher-intensity LTPA with metabolic and inflammatory variables. BMI and waist circumference negatively correlated with both lower- and higher-intensity LTPA after multivariate adjustments for age, sex, duration of diabetes, current smoking, current drinking, energy intake, cardiovascular diseases, depressive symptoms, treatment of diabetes, and higher (lower)-intensity LTPA. After further adjustment with BMI, waist circumference negatively correlated with higher-intensity LTPA but not with lower-intensity LTPA. HbA_1c_ and HOMA2-IR significantly correlated with only higher-intensity LTPA. These associations with higher-intensity LTPA remained significant after further adjustment with BMI. Triglyceride, HDL cholesterol, and HS-CRP significantly correlated with both lower- and higher-intensity LTPA after multivariate adjustments. These associations remained significant after further adjustment with BMI, except the association between HS-CRP and lower-intensity LTPA (p = 0.054). Furthermore, we analyzed the effects of the interaction between T-LTPA and the percentage of higher-intensity LTPA per T-LTPA and found that intensity differences were statistically significant for HbA_1c_ and HOMA2-IR (*P*_interaction_: 0.04 and 0.02, respectively), but not for waist circumference, triglyceride, HDL cholesterol, or HS-CRP (*P*_interaction_: 0.12, 0.93, 0.77, and 0.77, respectively).

**Table 3:** Multiple regression analysis of lower- or higher-intensity LTPA with metabolic and inflammatory variables.

		Lower-intensity LTPA (MET h/w)		Higher-intensity LTPA (MET h/w)	
		Regression coefficient	p value	Regression coefficient	p value
		(per 1 MET h/w increase)		(per 1 MET h/w increase)	
BMI (kg/m^2^)	Model 1	−0.0194	<0.001	−0.0176	<0.001
	Model 2+higher (lower)-intensity LTPA	−0.0195	<0.001	−0.0164	<0.001
Waist circumference (cm)	Model 1	−0.0461	<0.001	−0.0748	<0.001
	Model 2+higher (lower)-intensity LTPA	−0.0449	<0.001	−0.0679	<0.001
	+BMI	−0.0013	ns	−0.0313	<0.001
HbA1c (%) (mmol/mol)	Model 1	−0.0003 (−0.0033)	ns	−0.0005 (−0.0055)	<0.001
	Model 2+higher (lower)-intensity LTPA	−0.0002 (−0.0022)	ns	−0.0003 (−0.0033)	0.003
	+BMI	−0.0001 (−0.0011)	ns	−0.0003 (−0.0033)	0.027
HOMA2-IR	Model 1	−0.0016	0.069	−0.0038	<0.001
	Model 2+higher (lower)-intensity LTPA	−0.0015	0.092	−0.0034	<0.001
	+BMI	−0.0006	ns	−0.0026	<0.001
Triglyceride (mmol/l)	Model 1	−0.0024	<0.001	−0.0030	<0.001
	Model 2+higher (lower)-intensity LTPA	−0.0022	0.001	−0.0027	<0.001
	+BMI	−0.0015	0.016	−0.0021	<0.001
HDL-cholesterol (mmol/l)	Model 1	0.0020	<0.001	0.0027	<0.001
	Model 2+higher (lower)-intensity LTPA	0.0019	<0.001	0.0024	<0.001
	+BMI	0.0014	0.003	0.0020	<0.001
HS-CRP (mg/l)	Model 1	−0.0053	0.002	−0.0062	<0.001
	Model 2+higher (lower)-intensity LTPA	−0.0051	0.003	−0.0054	<0.001
	+BMI	−0.0032	0.054	−0.0039	0.002

Lower- and higher-intensity was defined as <3.6 METs (mean of study participants) and ≥3.6 METs, respectively.

Abbreviations: LTPA, leisure-time physical activity; METs, metabolic equivalents; BMI, body mass index; HbA_1c_, hemoglobin A_1c_; HOMA2-IR, homeostasis model assessment 2 of insulin resistance; HDL, high density lipoprotein; HS-CRP, high sensitivity C-reactive protein.

Model 1, adjusted for age and sex; Model 2, adjusted for age, sex, duration of diabetes, current smoking, current drinking, energy intake, cardiovascular diseases, depressive symptoms and treatment of diabetes.

## Discussion

The present study demonstrated that: (1) T-LTPA was dose-dependently associated with BMI, waist circumference, HbA_1c_, fasting plasma glucose, HOMA2-IR, triglyceride, HDL cholesterol, HS-CRP, and prevalence of metabolic syndrome; (2) T-LTPA was inversely associated with HbA_1c_ in non-obese participants but not in obese participants after multivariate adjustment; (3) higher-intensity LTPA (≥3.6 METs) was associated with HbA_1c_ and HOMA2-IR, whereas lower-intensity LTPA was not. Although there are many intervention trials using exercise at mostly moderate-to-high intensity PA in diabetic patients, epidemiological evidence regarding exercise as a treatment modality in real-world settings is scarce in Asian type 2 diabetic patients to the best of the authors' knowledge [26]–[28].

In the present study, the amounts of T-LTPA from which clinical variables significantly improved as compared with O1 (mean: 0.1 METs·h/w) were O3 (mean: 7.7 METs·h/w) for BMI, waist circumference, and HS-CRP, O4 (mean: 11.9 METs·h/w) for HOMA2-IR, HDL cholesterol, and prevalence of metabolic syndrome, and O5 (mean: 15.9 METs·h/w) for triglyceride and O6 (mean: 23.1 METs·h/w) for HbA_1c_, respectively. The amount recommended by the joint position statement of the ADA and ACSM in type 2 diabetic patients is 150 min/w of aerobic exercise of moderate-to-vigorous intensity [1]. When moderate intensity, defined as 3–6 METs (mean: 4.5 METs), is used, the recommended amount is converted into 7.5–15.0 METs·h/w (mean: 11.3 METs·h/w), corresponding to O3–O5 (O4) in our study. In addition, the joint position statement suggested that exercise may result in a small reduction in LDL cholesterol with no change in triglyceride or HDL cholesterol and slightly lower systolic blood pressure [1]. In the present study, however, LDL cholesterol or blood pressure did not differ according to T-LTPA, whereas triglyceride and HDL cholesterol dose-dependently improved. Dose-effect relationships between PA and clinical variables have been reported by Di Loreto *et al.* [29] who studied the effect of different amounts of increased PA in 179 type 2 diabetic patients. HbA_1c_, triglyceride, and blood pressure improved from the group mean of 17.1 METs·h/w and body weight, waist circumference, HDL cholesterol, and LDL cholesterol improved from the group mean of 27.0 METs·h/w. In the Italian Diabetes and Exercise Study, however, no clear dose-dependent effects except for BMI and waist circumference were observed [30]. In a recent meta-analysis [9], the mean decline in HbA_1c_ with structured exercise training was 0.67% (7.3 mmol/mol), whereas the difference in HbA_1c_ between O1 and O8 in our study was only 0.2% (2.2 mmol/mol). These conflicting results may be explained by differences in study design (intervention *vs.* observational study), methods in assessing PA, ethnicity [31] and so on.

Although exercise is a cornerstone of treatment in type 2 diabetic patients, its effect on glycemic control and cardiovascular risk factors has not been confirmed in Asians, who are typically non-obese. In this Japanese study (mean BMI: 23.8 kg/m^2^), increased T-LTPA was associated with improvement in glycemic control and cardiovascular risk factors, except for blood pressure and LDL cholesterol. When stratified by the presence of obesity, T-LTPA was not significantly associated with HbA_1c_ in obese participants after multivariate adjustment (Table 2). This is in contrast with a prevention study of Korean people (mean BMI: 25.0 kg/m^2^), which showed that regular exercise (300 min/w) of moderate-intensity prevented the development of type 2 diabetes in those with BMI≥25 kg/m^2^ but not with BMI<25.0 kg/m^2^ [32]. The disagreement may be because of differences in the study designs, i.e., observational *vs.* intervention study. In addition, the lack of an association between HbA_1c_ and T-LTPA seen in obese participants may be because of the smaller amounts of T-LTPA, especially higher-intensity LTPA, in obese participants compared with non-obese participants (T-LTPA: 15.7±18.0 METs·h/w *vs*. 20.3±19.3 METs·h/w, p<0.001; higher-intensity LTPA: 9.2±13.7 METs·h/w *vs.* 12.9±15.8 METs·h/w, p<0.001). As shown in Table 3, higher-intensity LTPA was associated with HbA_1c_ independent of BMI, but lower-intensity LTPA was not. The relationship between training intensity and improvement in glycemic control has been controversial. Umpierre *et al.* [4] reported that the frequency of aerobic exercise was associated with the reduction in HbA_1c_, but the intensity showed no significant association with changes in HbA_1c_ by meta-regression analysis of randomized clinical trials. In the Italian Diabetes and Exercise Study, the exercise effect on glycemic control and cardiovascular risk factors was prospectively compared between low-to-moderate and moderate-to-high intensity under similar amounts of total PA for 12 months [33]. Balducci *et al.* [33] reported that supervised moderate-to-high intensity exercise was marginally but significantly associated with better HbA_1c_, triglyceride, and total cholesterol levels. In the present study, the interaction between T-LTPA and the percentage of higher-intensity LTPA per T-LTPA was significant on HbA_1c_ and HOMA2-IR, suggesting the role of the intensity of PA in the regulation of glycemia and insulin action, at least in Japanese type 2 diabetic patients who are leaner than Western patients.

We found that the mean intensity of LTPA was 3.6 METs, the proportion of participants with low-intensity LTPA (<3.0 METs) was 6.0%, and the proportion of those with vigorous-intensity LTPA (≥6.0 METs) was 10.2% in type 2 diabetic patients attending diabetes special clinics in Japan. Therefore, most participants were considered to practice moderate-intensity LTPA (3.0–6.0 METs) as their non-pharmacological treatment. However, the amount of slightly lower-intensity LTPA (3.0–3.6 METs) was not associated with HbA_1c_ or HOMA2-IR by multiple regression analysis (data not shown), whereas the amount of slightly higher-intensity LTPA (3.6–6.0 METs) was significantly associated with them (HbA_1c_: β = −0.0004, p = 0.01 in model 2+lower-intensity LTPA; β = −0.0003, p = 0.03 in model 2+lower-intensity LTPA+BMI; HOMA2-IR: β = −0.0033, p<0.001 in model 2+lower-intensity LTPA; β = −0.0018, p = 0.001 in model 2+lower-intensity LTPA+BMI). These findings suggest that higher-intensity PA (for example, brisk walking, METs 3.8) may be required to improve glycemic control even in the range of moderate intensity (3.0–6.0 METs) of PA.

The anti-inflammatory effect of PA has been demonstrated in exercise intervention studies in type 2 diabetic patients [34]. The mechanisms behind the suppression of HS-CRP by PA may include: (1) the amelioration of insulin resistance induced by an exercise intensity-dependent release of interleukin-6 from muscle fibers [35]; and (2) enhanced expression of adiponectin receptor, despite unchanged serum adiponectin levels [36]. Chronic low-grade inflammation reflects the state of enhanced oxidative stress and insulin resistance, which may contribute to the development and progression of diabetic complications. Measures leading to a reduction in HS-CRP may be beneficial in preventing diabetic complications. Recently, Kodama *et al.* [3] reported that the risks of all-cause mortality and cardiovascular disease were exponentially suppressed with increasing habitual PA by a meta-analysis of cohort studies without exercise intervention. All-cause mortality and cardiovascular disease were reduced with medium amounts of LTPA (8.3 METs·h/w) corresponding to our O3 by 11.2% and 9.3%, respectively, and at high amounts of LTPA (16.7 METs·h/w) corresponding to our O5 by 21.2% and 17.9%, respectively. The present study showed that the amount of T-LTPA required to reduce HS-CRP was lower than that required to reduce HbA_1c_ (O3 *vs.* O6).

The strengths of the study are: (1) the sample size is relatively large (n = 4,870); (2) PA is quantified using a universal MET score in real-world settings [19]; and (3) confounding factors include dietary data and depressive symptoms. The limitations of the study are: (1) we did not measure occupational PA, although most participants seemed to have retired from work because their mean age was 65 years. We performed stratified analysis between ≥65 and <65 years of age, and the association between T-LTPA and glycemic control and CVD risk factors did not differ in those of ≥65 and <65 years of age (interaction p for the age of 65 years and T-LTPA in the age- and sex-adjusted model was 0.167 for BMI, 0.947 for waist circumference, 0.070 for HbA_1c_, 0.420 for HOMA2-IR, 0.832 for triglyceride, 0.315 for HDL cholesterol and 0.704 for HS-CRP, respectively); (2) practising PA may have been restricted because of medical reasons, such as severe retinopathy or arthritis. Although physical fitness was not assessed during recruitment for the study, participants seemed to be active enough to be able to attend the out-patient department regularly; (3) PA was assessed using a self-reported questionnaire, which may have resulted in an overestimation. In addition, participants may be physically less active in the winter time. However, the time of year did not significantly affect the T-LTPA because of the warm climate of our prefecture (18.1±17.7 METs·h/w in the spring, 18.5±19.5 METs·h/w in the summer, 19.6±18.7 METs·h/w in the fall, 20.2±20.8 METs·h/w in the winter, ns); (4) we cannot show any cause-and-effect relationships because of the cross-sectional design of our study; and (5) there may be other confounding factors besides those evaluated.

In conclusion, we demonstrated clear dose-dependent relationships between T-LTPA and glycemic control and some cardiovascular risk factors in Japanese type 2 diabetic patients. However, the amount of T-LTPA required to lower HbA_1c_ appeared to be greater than that required to improve other cardiovascular risk factors in our cohort. In addition, higher-intensity LTPA may be more appropriate even in the range of moderate-intensity PA recommended for diabetes treatment. From the results of this study, it is suggested that brisk walking more than 2 hours a week may improve the health of Japanese type 2 diabetic patients. Although increased PA may be an effective treatment in Japanese type 2 diabetic patients as well as in Western patients, there may be some ethnic differences between them.
